# The N-terminus of apolipoprotein B mediates the interaction of atherogenic lipoproteins with endothelial cells

**DOI:** 10.1172/JCI190513

**Published:** 2026-04-23

**Authors:** Ainara G. Cabodevilla, Camila Calistru, Waqas Younis, Dimitris Nasias, Tse W.W. Ho, Narasimha Anaganti, Swati Valmiki, Sujith Rajan, Jana Gjini, Rufina Kore, Carmen Hannemann, Nicholas O. Davidson, Tomas Vaisar, Jenny E. Kanter, Karin E. Bornfeldt, Edward A. Fisher, Warren L. Lee, Tobias Madl, M. Mahmood Hussain, Ira J. Goldberg

**Affiliations:** 1 Holman Division of Endocrinology, Diabetes, and Metabolism, Department of Medicine, New York University Grossman School of Medicine, New York, New York, USA.; 2 Keenan Centre for Biomedical Research, St. Michael’s Hospital, and Department of Laboratory Medicine and Pathobiology, University of Toronto, Toronto, Canada.; 3 Department of Foundations of Medicine, Diabetes and Obesity Research Center, New York University Grossman Long Island School of Medicine, Mineola, New York, USA.; 4 Division of Cardiology and Cardiovascular Research Center, Department of Medicine, New York University Grossman School of Medicine, New York, New York, USA.; 5 Division of Gastroenterology, Washington University School of Medicine, St. Louis, Missouri, USA.; 6 Department of Medicine, Division of Metabolism, Endocrinology, and Nutrition and; 7 Department of Laboratory Medicine and Pathology, University of Washington, Seattle, Washington, USA.; 8 Integrative Structural Biology Research Unit, Division of Medicinal Chemistry, Otto Loewi Research Center, Medical University of Graz, and BioTechMed-Graz, Graz, Austria.

**Keywords:** Metabolism, Vascular biology, Cardiovascular disease

## Abstract

Apolipoprotein B–containing (APOB-containing) lipoproteins contribute to atherosclerosis by entering the arterial wall through the endothelial cell (EC) surface receptors scavenger receptor-BI (SR-BI) and activin receptor-like kinase 1 (ALK1). We used N-terminal fragments of APOB, molecular modeling, and site-directed mutagenesis to identify and block the binding of chylomicrons and LDL to these receptors in cells and mice. We discovered that different APOB regions interact with SR-BI and ALK1 expressed on ECs. APOB48 lipoproteins were only internalized by SR-BI. A fragment of APOB comprising 18% of the N-terminal sequence, APOB18, reduced the uptake and transport of both chylomicrons and LDL by ECs, whereas a shorter fragment, APOB12, only blocked ALK1-mediated uptake of APOB100-containing lipoproteins. Importantly, overexpressing APOB18 decreased atherosclerosis in hypercholesterolemic mice. These findings identify the N-terminal region of APOB as the cause of atherosclerosis and illustrate an approach to treating or preventing vascular disease.

## Abstract 2

We identified a region in apolipoprotein B critical for atherosclerosis development and generated a small peptide that reduces atherosclerosis in mice.

## Introduction

Accumulation of cholesterol within the arterial wall is the cause and pathological signature of atherosclerosis. This occurs due to the movement of circulating lipoproteins containing the structural protein apolipoprotein B (APOB) across the arterial endothelial cell (EC) barrier (1). A large 550 kDa form of APOB, termed APOB100, is found in LDL, whereas chylomicrons and their remnants contain only the N-terminal 48% of APOB (termed APOB48). High circulating concentrations of lipoproteins containing either size of APOB cause atherosclerosis (2). What makes APOB lipoproteins atherogenic? APOB lipoproteins deliver triglyceride (TG) and cholesterol to various cells in the body by interacting with lipolysis enzymes and lipoprotein receptors. However, when their plasma levels increase, these lipoproteins accumulate in the subendothelial space, in part due to their binding to extracellular matrix proteins such as proteoglycans (3). Subsequently, cholesterol and other lipids, particularly phospholipids, may be oxidized to more toxic forms (4). These modified lipids or lipoproteins are internalized by macrophages leading to the development of foam cells (4). LDL particles are too large to freely cross the EC barrier. Their transcytosis is mediated by 2 receptors, activin receptor-like kinase 1 (ALK1) and scavenger receptor-BI (SR-BI) (reviewed in ref. 5). Knockout or inhibition of either of these receptors specifically in ECs reduces atherosclerosis progression (6, 7). SR-BI, but not ALK1, also internalizes APOB48-containing chylomicrons (8).

Aside from LDL, human data strongly implicate all APOB-containing lipoproteins in atherosclerosis development. Measurements of non-HDL cholesterol or APOB consistently correlate with cardiovascular disease (CVD) events better than LDL (9, 10). GWAS consistently identify genes associated with metabolism of TGs as markers of CVD risk (reviewed in ref. 11). Moreover, studies of postprandial lipemia (the increase in APOB lipoproteins after a meal) also associate with CVD risk (12). Thus, it is likely that all APOB-containing lipoproteins cause disease. The N- and C-terminal regions of APOB exist in close juxtaposition, as determined by both computer modeling (13) and cryo-electron microscopy (14). The site on APOB required for its interaction with the LDL receptor (LDLR) is in the C-terminal 30% of the molecule (15). This region of APOB, which is absent in chylomicrons, also contains residues that allow its interaction with proteoglycans (16). The N-terminal region of APOB in malondialdehyde-modified LDL was reported to contain a binding site for macrophage scavenger receptors (17). The sites required for uptake and transcytosis of APOB by ECs have not been defined. LDL uptake via ALK1 is not inhibited by APOB fragments containing the LDLR binding region (15), suggesting that the ALK1 binding region lies in either the N- or C-terminal nonlipophilic regions of APOB.

We aimed to determine the domains on APOB that bind to ALK1 and SR-BI and that are required for the transcytosis of APOB lipoproteins across the EC barrier. APOB contains large hydrophobic β-sheet domains that allow it to associate with nonpolar lipids such as cholesteryl esters and TGs (18). Although the secretion of APOB100 and APOB48 requires lipidation, mediated by microsomal TG transfer protein (MTP) (19), the N-terminal 18% of APOB contains hydrophilic domains and is secreted independently of MTP (20). Because the N-terminal region is common to all atherogenic lipoproteins, we hypothesized that APOB18 contains the binding sites to allow EC uptake and transcytosis of all APOB-containing lipoproteins. To test this, we used N-terminal fragments of APOB to competitively block these receptors and assessed how this affected uptake of APOB48 and APOB100 lipoproteins. We then used adenoviral overexpression to reduce uptake of APOB-containing lipoproteins by ECs and the development of atherosclerosis in LDLR-knockout (*Ldlr*^–/–^) mice.

## Results

### N-terminal APOB fragments inhibit APOB lipoprotein uptake by ECs.

**Figure 1:**
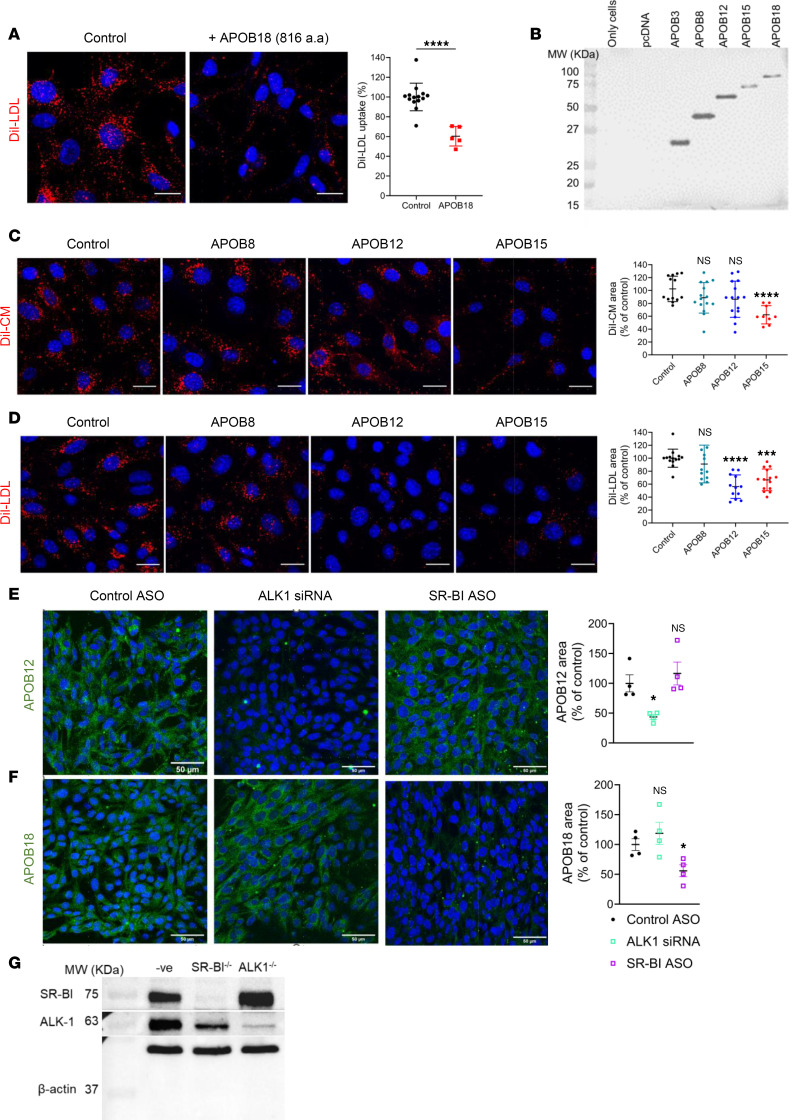
EC uptake of APOB100/APOB48 chylomicrons and LDL. Human aortic ECs were deprived of serum overnight, then incubated with DiI-labeled chylomicrons (4 mg/dL) or LDL (2.5 mg/dL) in serum-free medium for 30 minutes. (**A**) APOB18 significantly inhibits EC uptake of DiI-LDL. ****P < 0.0001, unpaired 2-tailed Student’s *t* test. (**B**) Western blot of C-terminal truncated APOB fragments used in competition studies. (**C**) APOB15, but not APOB9 or APOB12, inhibits uptake of APOB48-containing chylomicrons. (**D**) APOB12 and APOB15, but not APOB9, inhibits uptake of APOB100-containing LDL. Scale bars: 20 µm (**A**, **C**, and **D**). ***P < 0.0001, ****P < 0.00001, 1-way ANOVA followed by Dunnett’s post hoc multiple-comparison test against control in **C** and **D**. (**E**) Knockdown of ALK1, but not SR-BI, significantly reduces association of APOB12 to the surface of ECs. Scale bars: 50 μm. (**F**) Knockdown of SR-BI, but not ALK1, significantly reduces association of APOB18 to the surface of ECs. Scale bars: 50 μm. *P < 0.01, 1-way ANOVA followed by Dunnett’s post hoc multiple-comparison test against control. (**G**) Representative Western blot of receptor knockdown efficiency following treatment with ALK1 siRNA or SR-BI ASO. All images shown in this figure are representative of at least 4 independent experiments performed with 3 biological replicates. At least 4,000 cells per condition were analyzed.

We previously showed that SR-BI–mediated chylomicron uptake was inhibited by a fragment of APOB containing the 18% N-terminal sequence of the molecule (APOB18), suggesting that this region is required for APOB binding to EC receptor SR-BI (8). For this reason, we assessed whether APOB18 would also inhibit LDL uptake. Although ECs express low levels of LDLR, 24 hours before treatment, ECs cultured in glass coverslips were switched to media enriched in human LDL (25 μg/mL) to ensure complete LDLR depletion (15). ECs were then washed thoroughly with PBS and treated with serum-free media containing 2.5 μg/mL DiI-labeled LDL in the absence or presence of APOB18 for 30 minutes. Cells were then washed with PBS and fixed with 10% formalin, and nuclei were stained with DAPI. DiI-LDL uptake was assessed by confocal microscopy, and quantification of the area occupied by DiI was performed using ImageJ/Fiji. As shown in Figure 1A, competition with APOB18 led to a significant (39.72% ± 9.94%) reduction of DiI-LDL uptake compared with control.

To further pinpoint the region in APOB responsible for EC receptor binding, we used smaller fragments of APOB to compete DiI-labeled chylomicron and LDL uptake (Figure 1B). As shown in Figure 1C, APOB15, but not APOB12 or APOB8, blocked chylomicron uptake by ECs. This suggested that a region between APOB12 and APOB15 interacts with SR-BI and that APOB12 lacks the motif required to block this receptor. In contrast, APOB12 blocked the uptake of DiI-LDL, which was also inhibited by competition with APOB15 but not APOB8 (Figure 1D). This implied that the N-terminal region between APOB8 and APOB12 contains the ALK1 binding site and that this site differs from that required for chylomicron uptake.

To directly assess binding of APOB fragments to EC receptors, we performed membrane association studies in ECs treated with SR-BI antisense oligonucleotide (ASO) or ALK1 siRNA. As shown in Figure 1E, knockdown of ALK1 but not SR-BI significantly reduced association of FLAG-tagged APOB12 to the surface of ECs. In contrast, binding of APOB18 to EC membranes was significantly reduced by knockdown of SR-BI but not that of ALK1 (Figure 1F).

Knockdown of the receptors is shown in Figure 1G. As previously reported (21), SR-BI ASO also led to a reduction in ALK1 expression, but SR-BI expression was not reduced by treatment with ALK1 siRNA.

### APOB length affects its interaction with EC receptors.

If chylomicrons and LDL contain identical N-terminal regions, why does APOB12 and ALK1 knockdown (8) not affect chylomicron uptake? We hypothesized that this is due to differences in either the size of the lipoproteins — chylomicrons, with sizes ranging from 75 to 450 nm (22), are much larger than LDL — or the length of the APOB. The N-terminal region of APOB forms a loop that brings C-terminal epitopes in proximity to the N terminus, and Flood et al. reported that removal of the C-terminal portion of APOB exposes N-terminal proteoglycan binding sites (23). More recent structural analyses of APOB are consistent with a model suggesting molecular interactions between the N- and C-terminal APOB regions (13, 14).

**Figure 2:**
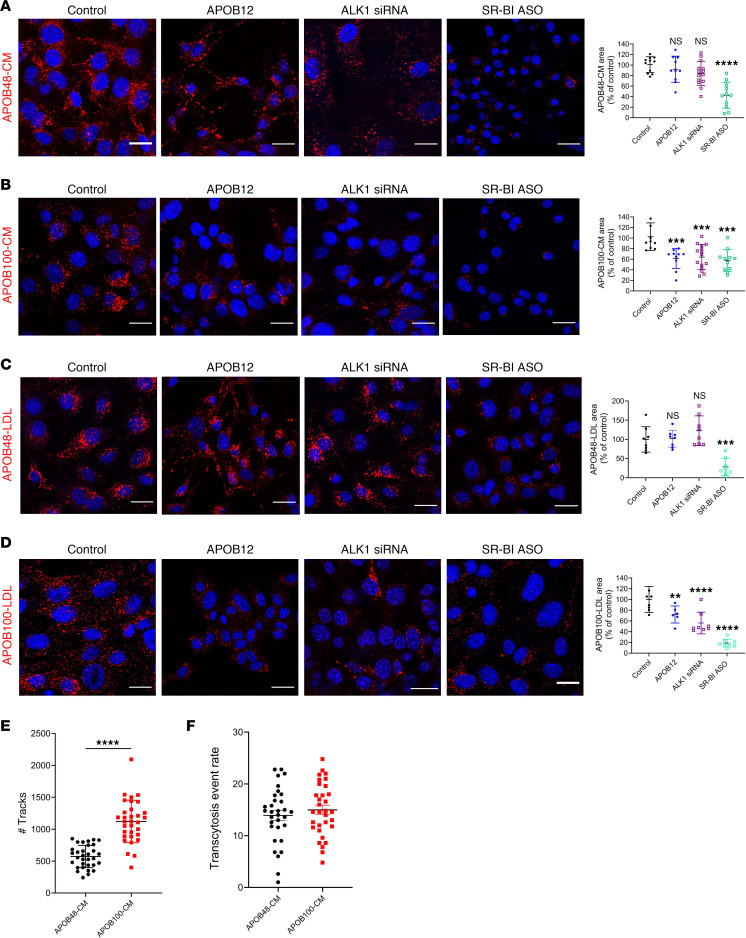
ALK1-mediated lipoprotein uptake requires APOB100. (**A**) Uptake of chylomicrons obtained from APOB48-only mice is inhibited by SR-BI knockdown (KD) with ASO, but not by APOB12 or ALK1 KD with siRNA. (**B**) Uptake of APOB100 chylomicrons obtained from *Apobec1*^–/–^ mice is inhibited by competition with APOB12, as well as KD of both SR-BI and ALK1. (**C**) As with chylomicrons, uptake of APOB48-LDL is inhibited by SR-BI KD but not ALK1 KD or APOB12. (**D**) Uptake of APOB100 LDL is inhibited by competition with APOB12, as well as KD of both SR-BI and ALK1. Scale bars: 10 μm (**A**–**D**). **P < 0.001, ***P < 0.0001, ****P < 0.00001, 1-way ANOVA followed by Dunnett’s post hoc multiple-comparison test against control. (**E**) Vesicles bearing APOB100 chylomicrons are transported to the base of the cell at a significantly higher rate than vesicles bearing APOB48 chylomicrons. (**F**) The transcytosis event rate of APOB100 and APOB48 chylomicrons is not significantly different. ****P < 0.00001, unpaired 2-tailed Student’s *t* test. Results are shown as scatterplots (mean ± SD).

To assess whether the C-terminal region is required for the uptake of APOB lipoproteins via ALK1, we isolated lipoproteins from mice only expressing APOB48 (2) and from APOBEC1-deficient mice that only produce APOB100 (24). Although APOB100-carrying chylomicrons are not a physiological lipoprotein species in mammals, these particles provided a useful experimental model to assess the contribution of the APOB C-terminal domain to EC uptake. As expected from our previous studies (8), uptake of APOB48 chylomicrons was inhibited by SR-BI ASO. However, neither ALK1 siRNA nor APOB12 reduced EC uptake of APOB48 chylomicrons (Figure 2A). In contrast, uptake of APOB100 chylomicrons by ECs was inhibited by APOB12 as well as by SR-BI and ALK1 knockdown (Figure 2B). These studies indicate that APOB100 chylomicrons interact with both receptors, whereas the region required for ALK1 binding appears to be obscured in APOB48-containing lipoproteins.

Next, we determined the receptors required for uptake of APOB48 and APOB100 LDL. Similar to APOB48 chylomicrons, APOB48 LDL uptake by ECs was blocked by SR-BI ASO but not ALK1 siRNA or APOB12 (Figure 2C). As expected, uptake of APOB100 LDL was inhibited by APOB12 and knockdown of both receptors (Figure 2D). These data suggest that distinct motifs in the N-terminal region of APOB interact with the 2 receptors and that the disposition of APOB100 and APOB48 on lipoproteins determines receptor binding independent of lipoprotein size.

### APOB100 mediates lipoprotein transcytosis.

We previously showed that endothelial SR-BI–mediated APOB48 chylomicron uptake results in intracellular chylomicron hydrolysis in the lysosomal compartment (8). In contrast, APOB100-containing LDL undergoes endothelial transcytosis, which is mediated by both SR-BI and ALK1 (7, 15). To assess whether the presence of the entire APOB100 would result in chylomicron transcytosis, we performed total internal reflection fluorescence (TIRF) microscopy to visualize chylomicrons at the basolateral membrane and enumerate transcytosis events (25). Within minutes of allowing binding to the apical endothelial surface, we observed significantly more APOB100 chylomicrons at the base of the cell compared with APOB48 chylomicrons, consistent with increased internalization and traffic toward the bottom of the cell (Figure 2E). However, exocytosis rates of DiI from both particles were similar (Figure 2F).

### APOB molecular modeling of SR-BI and ALK1 ligand sites.

**Figure 3:**
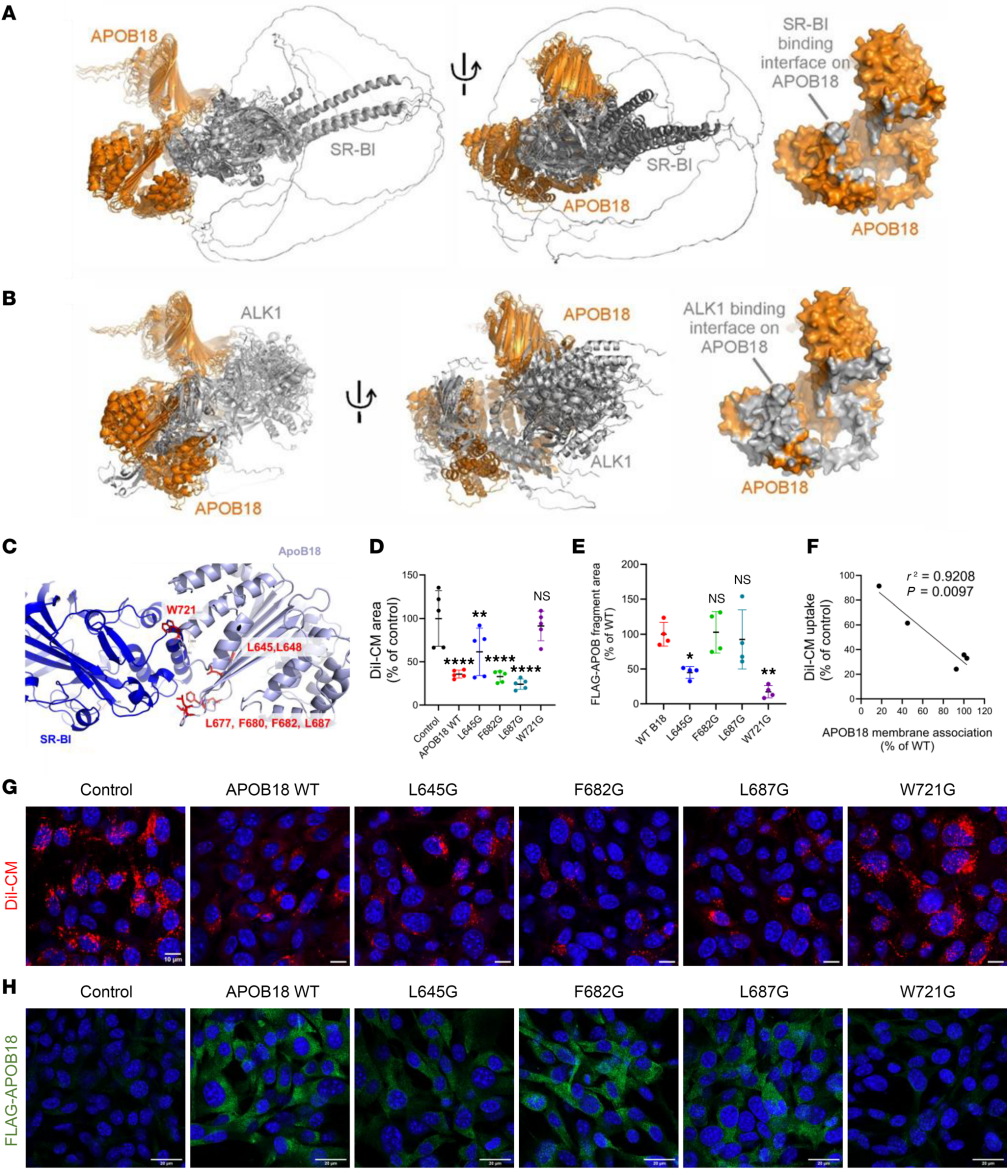
Molecular modeling and mutational studies of APOB18 complexes. (**A** and **B**) AlphaFold 2 predictions of the APOB18–SR-BI (**A**) and APOB18-ALK1 (**B**) complex structures. For all AlphaFold 2 predictions, an overlay of 5 independent calculations is shown. The binding interfaces of SR-BI and ALK1 on the surface of APOB18 are visualized by coloring all residues within APOB18, which are within 5 Å distance of SR-BI and ALK1. Note that the ALK1 binding interface appears more extensive due to the nonconvergence of the structural models. (**C**) Display of surface-exposed hydrophobic residues selected for mutational studies. (**D**) ECs were deprived of serum overnight, then incubated with DiI-labeled chylomicrons (4 mg/dL) in serum-free medium and in the presence of control, WT, or mutant APOB18, as indicated. DiI-chylomicron uptake was strongly and similarly inhibited by WT, L682, and L687 APOB18 (≈80% inhibition). (**E**) In contrast, L645G APOB18 was less potent in inhibiting CM uptake, and W721G mutation completely blunted CM uptake inhibition. (**F**) The inhibitory efficiency of mutant APOB18 fragments significantly correlates with their ability to associate with the EC surface. (**G** and **H**) Representative images of 4 independent competition (**G**) and membrane association studies (**H**), each performed with 3 biological replicates. Scale bars, 10 μm (**G**), 20 μm (**H**). *P < 0.01, **P < 0.001, ****P < 0.00001, 1-way ANOVA followed by Dunnett’s post hoc multiple-comparison test against control.

We previously reported that treatment with heparin, which competes for and releases lipoproteins from glycosaminoglycan binding, did not inhibit EC uptake of chylomicrons (8). This suggested that uptake was not mediated by glycosaminoglycan binding and was consistent with the possibility that interactions between APOB and EC receptors involved hydrophobic contacts, although other modes of interaction could not be excluded. To identify hydrophobic patches that could be involved in the interactions between APOB18 and different receptors, we performed molecular modeling using AlphaFold 2 and the sequences of APOB18 and SR-BI as input. Our analyses yielded structural models with a converging surface area of APOB18 contacted by SR-BI (Figure 3A). Similar calculations using ALK1 as ligand yielded nonconverged interaction sites partially overlapping with the SR-BI binding site (Figure 3B). A summary of the AlphaFold predictions for the binding of APOB18 with SR-B1 and ALK-1 are summarized in Supplemental Figure 1, A and B, respectively (supplemental material available online with this article; https://doi.org/10.1172/JCI190513DS1). Inspection of the binding interface revealed that the site is enriched in hydrophobic residues and that several hydrophobic residues were exposed to the surface (Figure 3C). To identify critical residues in APOB18 for the inhibition of lipoprotein uptake by ECs, we performed site-directed mutagenesis to replace different surface-exposed hydrophobic residues located in APOB18 loops contacting SR-BI and ALK1 in the AlphaFold 2 models with glycine. To assess the effects of the loss of surface-exposed hydrophobic residues, we performed competition and surface association studies with the resulting mutant APOB18 fragments (Figure 3, D–H). As before, WT APOB18 significantly inhibited the uptake of DiI-chylomicrons by ECs. Mutagenesis of residues F682 and L687 to glycine had no effect on the inhibitory capacity of APOB18. In contrast, the inhibitory effect of APOB18 was substantially reduced after mutagenesis of L645 and completely precluded by mutagenesis of W721 (Figure 3D, representative confocal microscopy images are shown in Figure 3G). Similar effects were observed in competition with DiI-labeled LDL (Supplemental Figure 2A, quantification of 4 independent experiments is shown in Supplemental Figure 2B). Consistently, there was no significant difference in surface binding of L682G or L687G compared with WT APOB18, whereas association with ECs was significantly reduced in L645G and completely blunted in W721G (Figure 3E, representative images of 4 independent experiments are shown in Figure 3H). The inhibitory efficiency of mutant APOB18 fragments strongly correlated with their ability to associate with the EC surface (Figure 3F). Although glycine substitutions could potentially cause generalized misfolding, the differing effects observed across multiple mutations within the same region make this an unlikely explanation. Overall, these studies indicate that W721 and L645 are critical residues involved in binding to EC receptors and support our model of APOB–SR-BI interaction.

### Overexpression of APOB18 inhibits aortic EC chylomicron uptake and lipid droplet formation in aortas.

**Figure 4:**
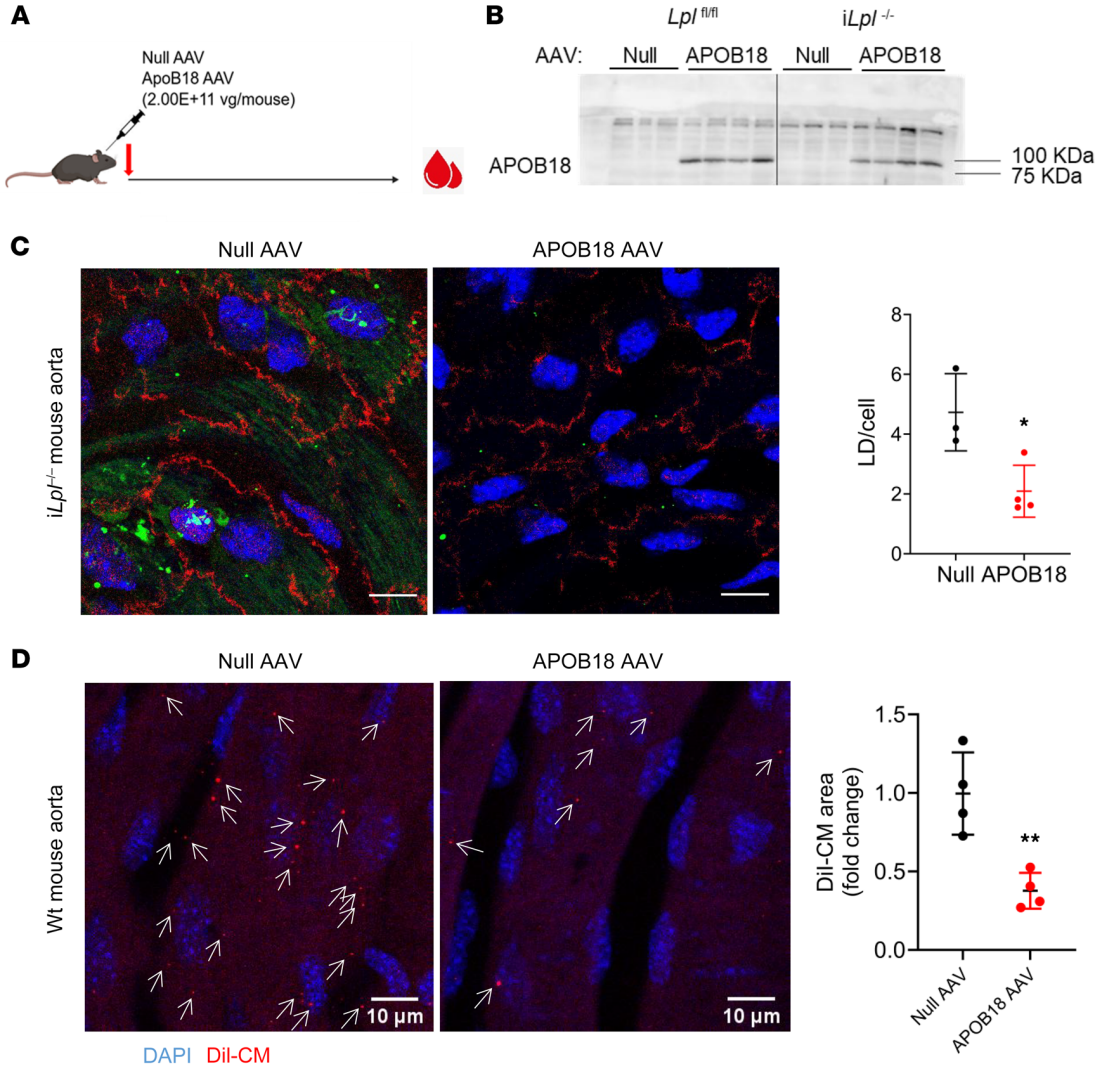
Treatment with APOB18-AAV inhibits aortic EC chylomicron uptake in vivo. (**A**) *Lpl*^fl/fl^ and i*Lpl*^–/–^ mice were injected intravenously with 2.00 × 10^11^ vg control (null) or APOB18 liver-targeted AAV8. (**B**) APOB18 was detected by immunoblot in the plasma of APOB18-AAV–treated mice 2 weeks after injection. (**C**) i*Lpl*^–/–^ mice treated with APOB18-AAV exhibit significantly fewer postprandial lipid droplets (green) in their aortic endothelium 180 minutes after a gavage with 200 μL olive oil. Scale bars: 10 μm. (**D**) Null and APOB18-AAV–treated *Lpl*^fl/fl^ mice were given DiI-labeled chylomicrons (red) by intravenous injection and euthanized 15 minutes later. Scale bars: 10 μm. Mice treated with APOB18-AAV exhibited significantly fewer chylomicrons in their aortic endothelium. *P < 0.01, **P < 0.001, unpaired 2-tailed Student’s *t* test.

During the postprandial period, aortic ECs develop lipid droplets (26). This is due to chylomicron uptake via SR-BI, and accumulation of aortic EC lipid droplets is exacerbated in lipoprotein lipase-deficient (LpL-deficient) mice (8) that have greater fasting and postprandial hypertriglyceridemia (27). To test whether APOB fragments inhibit chylomicron uptake by ECs in vivo, we transduced LpL-knockout mice with adeno-associated virus 8 (AAV8) for the expression of human APOB18 under the control of a liver-targeting thyroxin binding globulin promoter (Figure 4A). Plasma of APOB18-AAV–transduced mice had high levels of human APOB18. However, this did not alter circulating levels of APOB48 and APOB100 (Figure 4B). Three weeks after transduction, mice were gavaged with olive oil (10 mL/kg) and euthanized 3 hours later to assess postprandial lipid droplet accumulation in aortic ECs. Confocal microscopy imaging of en face aortas stained with neutral lipid dye BODIPY revealed that APOB18-AAV–treated mice had significantly reduced numbers of lipid droplets in the aortic endothelium (Figure 4C), suggesting that chylomicron uptake was reduced. To directly assess the impact of APOB18-AAV on aortic EC chylomicron uptake, we intravenously injected control and APOB18-AAV–expressing WT mice with DiI-labeled human chylomicrons. Aortic EC accumulation of DiI-chylomicrons was markedly reduced in mice treated with APOB18-AAV (Figure 4D). Thus, APOB18-AAV blocks EC uptake of chylomicrons in vivo.

### Expression of APOB18 in LDLR-knockout mice reduces atherosclerosis.

**Figure 5:**
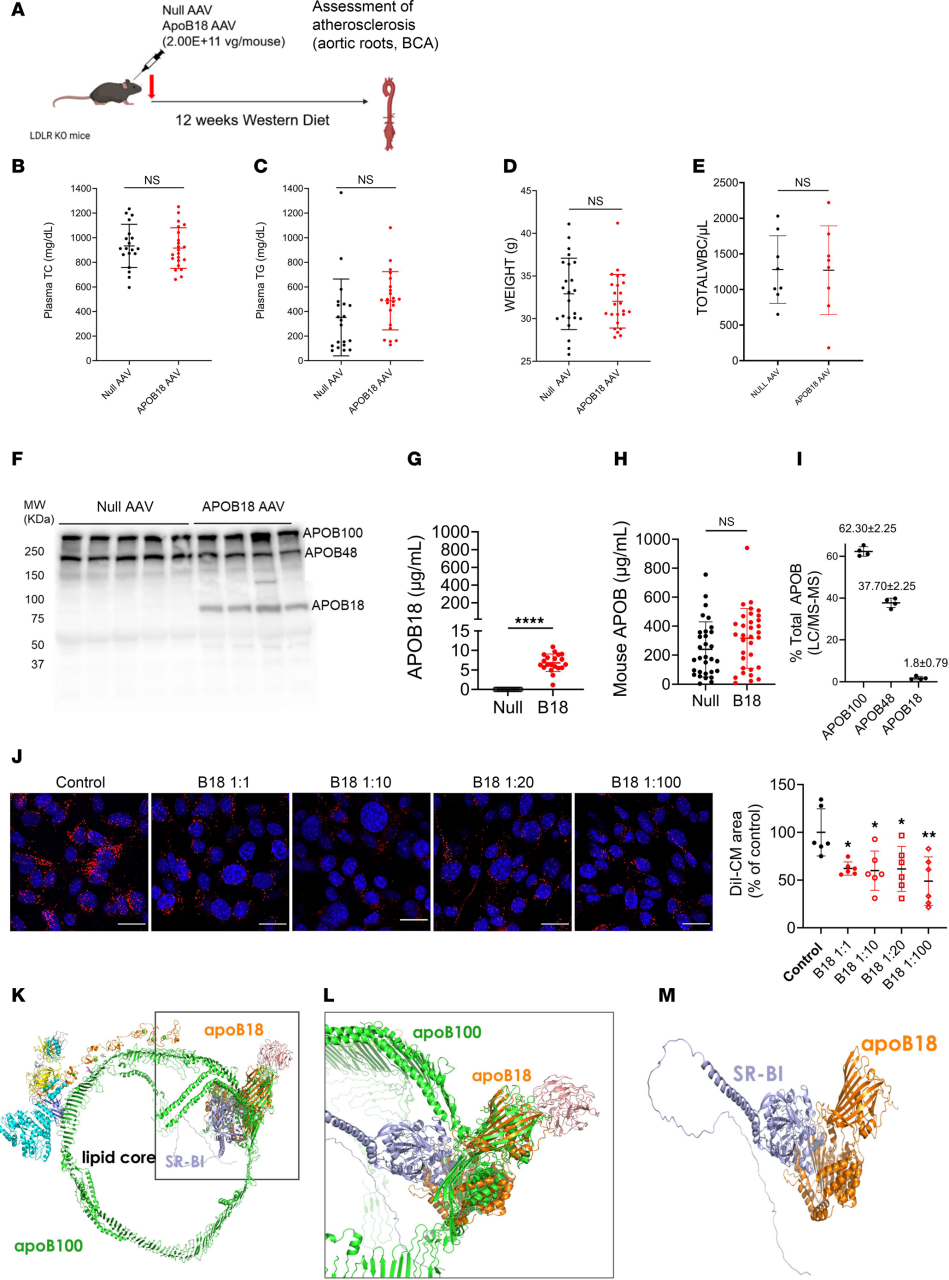
APOB18 blocks EC chylomicron uptake at low relative concentrations. (**A**) *Ldlr^–/–^* mice were injected intravenously with 2.00 × 10^11^ vg control (null) or APOB18-AAV and fed a western diet for 12 weeks. (**B**–**E**) Treatment with APOB18-AAV did not lead to significant changes in plasma cholesterol (**B**), TGs (**C**), BW (**D**), or WBC count (**E**). TC, total cholesterol. (**F**) Circulating levels of APOB18 appeared to be markedly lower than those of APOB48 or APOB100. (**G**) APOB18 was measured by ELISA using mouse monoclonal APOB antibody 1D1, which recognizes N-terminal amino acids 474–539 in human APOB. (**H**) Mouse APOB48 and APOB100 were measured using a commercial mouse APOB ELISA kit (Abcam, ab230932). The plasma concentration of APOB18 in mice treated with APOB18-AAV was approximately 40-fold lower than that of endogenous mouse APOB48 or APOB100. ****P < 0.00001, unpaired 2-tailed Student’s *t* test. (**I**) Plasma levels of APOB18, APOB48, and APOB100 were quantified by LC-MS/MS, and the relative abundance of each isoform was calculated as a percentage of total APOB. APOB18 comprised 1.8% ± 0.79% of total plasma APOB. (**J**) Competition with APOB18 inhibited chylomicron uptake at relative concentrations of 1:1, 1:10, 1:20, and 1:100. Scale bars: 20 µm. *P < 0.01, **P < 0.001, 1-way ANOVA followed by Dunnett’s post hoc multiple-comparison test against control. (**K**–**M**) Molecular modeling of the interaction of APOB100 in lipoproteins or APOB18 with SR-BI. When APOB is on lipoproteins, some of the APOB epitopes predicted to mediate binding to the receptor may be partially embedded in the lipid core (**K** and **L**). In APOB18, which is not lipidated, those epitopes are exposed, increasing its affinity for SR-BI (**M**).

Because APOB18 blocked both chylomicron and LDL uptake by ECs, we next tested whether treatment with APOB18-AAV would reduce atherosclerosis in *Ldlr*^–/–^ mice. The experimental plan is shown in Figure 5A. Eight-week-old mice were retro-orbitally injected with control (null) or APOB18-AAV and fed a western diet for 12 weeks; after which, plasma, aortic roots, and brachiocephalic arteries were collected for analysis. APOB18-AAV did not affect circulating levels of cholesterol (Figure 5B) and TG (Figure 5C). In addition to APOB, SR-BI binds HDL through high-affinity interactions with APOA1 at the HDL surface (28). We (8) and others (29–31) have shown that HDL strongly inhibits SR-BI–mediated uptake of APOB-containing lipoproteins, indicating that binding to HDL occurs with higher affinity. Consistently, treatment with APOB18-AAV did not alter circulating HDL-cholesterol or HDL-TG levels (Supplemental Figure 3A) nor did the virus have a significant impact on body weight (Figure 5D) or WBC count (Figure 5E) following 12 weeks on a western diet.

Figure 5F is a representative Western blot showing plasma levels of APOB100, APOB48, and APOB18 in mice treated with control or APOB18-AAV using a polyclonal antibody that recognizes epitopes in the N-terminus of both human and mouse APOB. Western blots of plasma from all mice treated with APOB18-AAV for these studies are shown in Supplemental Figure 3A. In all mice, circulating levels of APOB18 appeared to be markedly lower than those of APOB48 or APOB100. The use of a polyclonal antibody, characterized by heterogeneous epitope recognition, precluded reliable densitometric quantification. Therefore, we used mouse monoclonal APOB antibody 1D1, which recognizes N-terminal amino acids 474–539 in human APOB, as capture antibody for the detection of APOB18 by ELISA. Mouse APOB48 and APOB100 were measured using a commercial mouse APOB ELISA kit (Abcam, ab230932). The plasma concentration of APOB18 in mice treated with APOB18-AAV was 6.83 ± 2.23 μg/mL (Figure 5G), approximately 40-fold lower than that of endogenous mouse APOB48 (239.7 ± 189.5 μg/mL) or APOB100 (316.1 ± 205.3 μg/mL; Figure 5H). In addition, plasma levels of APOB18, APOB48, and APOB100 were independently quantified by liquid chromatography–tandem mass spectrometry (LC-MS/MS), and the relative abundance of each isoform was calculated as a percentage of total APOB (Figure 5I). According to this LC-MS/MS–based relative quantification, APOB18 represented 1.8% ± 0.79% of total plasma APOB in APOB18-AAV–treated mice, consistent with our concentration measurements.

To assess whether such low levels of APOB18 would be sufficient to inhibit EC chylomicron and LDL uptake, we performed titration studies using decreasing concentrations of APOB18. EC chylomicron uptake was inhibited by APOB18 in relative concentrations as low as 1:100 (Figure 5J), and we observed the same in competitions with LDL (Supplemental Figure 3B). To analyze this, we performed additional molecular modeling of the interaction of APOB100 in lipoproteins or APOB18 with SR-BI (Figure 5, K–M). When APOB is on lipoproteins, some of the APOB epitopes predicted to mediate binding to the receptor may be partially embedded in the lipid core (Figure 5, K and L). In APOB18, which is not lipidated, those epitopes are exposed, increasing its affinity for SR-BI (Figure 5M). Therefore, the relatively lower concentration of APOB18 in mouse plasma should not preclude inhibition of APOB-containing lipoproteins.

**Figure 6:**
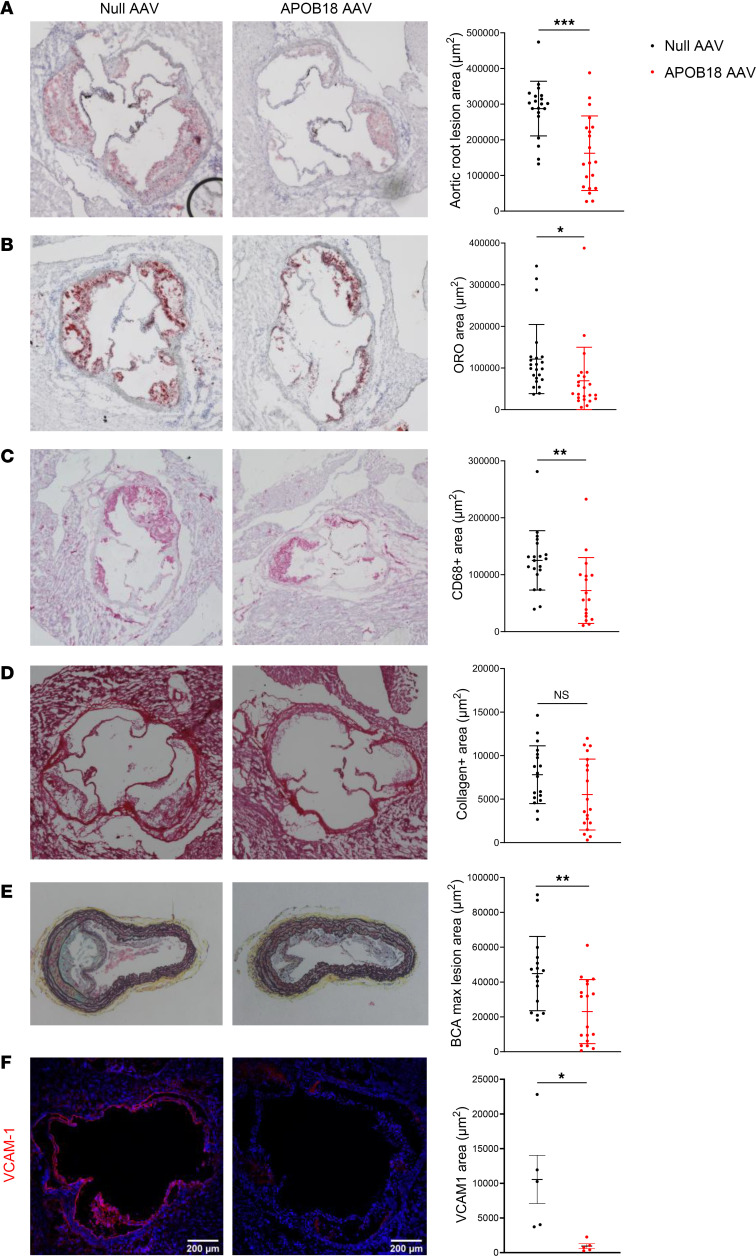
Treatment with APOB18-AAV reduces atherosclerosis. *Ldlr*^–/–^ mice were injected intravenously with 2.00 × 10^11^ vg control (null) or APOB18-AAV and fed a western diet for 12 weeks. Mice were then euthanized, and their aortic roots and BCA were harvested for assessment of atherosclerosis. (**A**) Mice treated with APOB18-AAV exhibited smaller lesions in their aortic roots. (**B**–**D**) Mice treated with APOB18-AAV exhibited a reduction in the area occupied by lipids (stained with Oil Red O [ORO]; **B**), macrophages (stained with CD68; **C**), and collagen (**D**). (**E**) Mice treated with APOB18-AAV exhibited significantly smaller lesions in their BCA. Lesion area in **A**–**E** was quantified from serial aortic root sections imaged at ×4 magnification. (**F**) Expression of VCAM-1 in the aortic roots was assessed in a subset of mice (5 per group) and was found to be significantly reduced in APOB18-AAV–treated mice. Scale bars: 200 μm. *P < 0.01, **P < 0.001, ***P < 0.0001, unpaired 2-tailed Student’s *t* test.

Consistently, atherosclerosis was reduced in the aortic root of APOB18-AAV–transduced mice fed a western diet for 12 weeks (Figure 6A). There was a reduction in the area occupied by lipids and macrophages (Figure 6, B and C) but no obvious changes in the lesion morphology as the percentage of reduction of these lesional components was similar. No changes were observed in the percentage of area occupied by collagen (Figure 6D). Brachiocephalic arteries of APOB18-AAV–transduced mice showed a similar reduction in lesion size (Figure 6E). We recently showed that chylomicron uptake by ECs in vitro induces the expression of a host of inflammatory genes, including VCAM1 and ICAM1 (32), which are also increased in the aortas of hyperchylomicronemia mice deficient in both LpL and LDLR (33). Immunohistochemical labeling showed that VCAM1 and ICAM1 were both significantly reduced in the aortic roots of APOB18-AAV–transduced mice (Figure 6F and Supplemental Figure 4). Therefore, APOB18, which competes for EC lipoprotein receptors, reduces atherogenesis.

## Discussion

The initial step in atherosclerosis development is the transfer of cholesterol-containing lipoproteins from circulation into the arterial wall. The intact EC barrier is impermeable to particles the size of lipoproteins. Therefore, entry of lipoprotein lipid into the subendothelial space must occur via nonspecific movement between or through ECs or via specific processes that mediate transcytosis of lipoproteins to the subendothelial space. Electron microscopy studies of atherosclerotic arteries document the presence of LDL within an intact EC lining (34), presumably reflecting cellular uptake of these particles. The receptors mediating this process have been described recently (5) and EC-specific deletion of either SR-BI or ALK1 reduces atherosclerosis (6, 7). In addition, we (8, 33) and others (35, 36) have shown that increased lipid content of ECs promotes inflammation, which in turn drives atherosclerosis. Here, we show that lipoprotein uptake via these receptors is through their interaction with specific regions of APOB within the N-terminal portion of the molecule. Moreover, the binding domains for SR-BI and ALK1 differ. Using this information, we overexpressed fragments of APOB in cells and mice. APOB18 inhibited chylomicron and LDL uptake by ECs in vitro and aortic uptake of chylomicrons in vivo. We then showed that APOB18-AAV also reduced atherosclerosis in *Ldlr*^–/–^ mice.

Our studies answer a long-standing question in atherosclerosis research: why are APOB-containing lipoproteins atherogenic? APOB is a 4,563–amino acid, approximately 550 kDa protein that is the major structural component of chylomicrons, VLDL, and LDL. Within the human gut and rodent gut and liver, a smaller version of this protein conventionally termed APOB48 is synthesized due to RNA editing that leads to termination of translation and loss of 52% of the C-terminal APOB protein (23). We show that APOB48-chylomicron and APOB100-LDL interact with endothelial receptors via different sites. APOB18 blocked both chylomicron and LDL uptake by ECs. However, APOB12 did not affect chylomicron uptake by SR-BI but did reduce LDL uptake by ALK1. Using several genetically modified mice to create APOB100 chylomicrons and APOB48 LDL, we show that APOB length, but importantly not lipoprotein size, determines receptor interaction. Lipoprotein binding to ALK1 requires the C-terminal region of APOB, perhaps because it allows exposure of a receptor-binding site. Thus, although APOB100 and APOB48 have an identical N-terminal region, the length of APOB, as has previously been suggested, likely affects the exposure of regions on this non–lipid-associated portion of the molecule (23).

The question then becomes, why does APOB18 inhibit the uptake of both chylomicrons and LDL by ECs? A simple explanation is that both receptor binding domains are exposed in the truncated fragment, whereas these motifs on lipoproteins are differentially exposed in LDL and chylomicrons, allowing recognition by different receptors. This hypothesis is supported by the structural modeling and older studies of the N-terminal region of APOB showing that the proximity to lipid, as would occur in lipoproteins, affects the N-terminal region configuration (37). This information suggests that synthetic peptides or their mimics at molar concentrations lower than that of APOB in lipoproteins can be used as therapeutic agents to reduce atherosclerosis without affecting plasma lipoprotein levels.

APOB has several charged regions that interact with proteoglycans, including a highly charged region in the vicinity of the LDLR binding portion of the molecule (23). Despite this, APOB48 lipoproteins that are missing this region have a similar affinity for heparin, as does APOB100 LDL (38). This appears to be because the C-terminal region of APOB affects the conformation of the N-terminal region and the exposure of proteoglycan-binding sites (23). By assessing the ability of APOB fragments to inhibit binding to ALK1 and SR-BI and molecular modeling, our data also suggest that the conformation of the N-terminal region of APOB is modified by the C-terminal region. This allows EC uptake of both APOB48 and APOB100 lipoproteins, albeit by different receptors. We confirmed the predicted SR-BI interaction sites by creating substitution mutants of hydrophobic amino acids and showing a loss of competition for chylomicron and LDL uptake by EC as well as reduced association of the mutated fragments to the EC surface.

The total number of APOB-containing lipoproteins correlates more strongly with development of cardiovascular events than measurements of LDL-cholesterol (reviewed in ref. 39). This suggests that TG-rich lipoproteins such as VLDL or chylomicron remnants are atherogenic. In humans, both VLDL and LDL contain APOB100. During the postprandial period and in some human diseases, significant numbers of APOB48 particles are also circulating (40). A large body of literature suggests that the level of postprandial lipemia is a risk factor for CVD in humans (reviewed in ref. 41), consistent with the hypothesis that APOB48 lipoproteins are atherogenic. Unlike humans, mice produce APOB48 in the liver and have higher circulating levels of APOB48 lipoproteins (42). We suspect that this is why deletion of either SR-BI (7) or ALK1 (6) reduces atherosclerosis in mice. Our data show that APOB48 and APOB100 lipoproteins are toxic to the arterial wall because their N-terminal region binds to EC receptors that allow uptake and transcytosis of atherogenic lipids.

In summary, atherosclerosis requires an initial uptake of APOB-containing lipoproteins by arterial ECs. This occurs via 2 receptors whose ligands, we show, are different residues in the N-terminal region of APOB. The exposure of these residues is affected by APOB length, which determines their affinity for the 2 different EC receptors. This is the reason that both APOB48 and APOB100 lipoproteins are atherogenic. Aside from establishing the mechanism of APOB uptake by EC lipoprotein receptors, our studies demonstrate that blocking these receptors using an N-terminal APOB fragment reduces atherosclerosis and provides an alternative approach to protecting the artery from elevated circulating APOB lipoprotein levels. This approach might be clinically useful in treating patients whose atherosclerosis progresses despite their use of lipid-lowering medications.

## Methods

### Sex as a biological variable.

Male mice were used to reduce variability associated with estrous cycle–related hormonal changes. We expect that our findings are likely to be relevant to more than one sex.

### Mice and diets.

LDLR-deficient (*Ldlr*^–/–^; B6.129S7-*Ldlr^tm1Her^*/J, stock no. 002207) and WT C57BL/6J mice (stock no. 000664) were obtained from The Jackson Laboratory. Global inducible LpL-knockout mice were generated by crossing floxed Lpl (*Lpl*^fl/fl^) mice with β-actin–driven, tamoxifen-inducible Cre (MerCreMer) transgenic mice (The Jackson Laboratory) to obtain the β-actin-MerCreMer/*Lpl*^fl/fl^ offspring designated as i*Lpl*^−/−^ mice, as previously described (43). Mice received i.p. injections of tamoxifen (Toronto Research Chemicals, T006000) in corn oil (Sigma-Aldrich, C8267) at a dose of 40 mg/kg BW/day 4 times, administered every other day. 12- to 18-week-old male mice were maintained in a temperature-controlled (25°C) facility with a 12-hour light/dark cycle. Mice were given free access to water and food, except when fasting blood specimens were obtained. Mice were either fed a rodent chow diet or an atherogenic western diet (Dyets, catalog 101977, 0.3% cholesterol), as indicated.

### Atherosclerotic lesion analysis.

Each anesthetized mouse was perfused with PBS. The aorta was then exposed, and fat was carefully cleaned under a binocular microscope. Pictures of the aortic arch and brachial cephalic artery (BCA) were taken using a camera fitted to the binocular microscope. The BCA was collected in 10% formalin, kept overnight at 4°C, and stored in 70% ethanol at 4°C for further processing. The root of the heart was cut and embedded in TissueTek OCT, frozen, and stored at –80°C. Serial sections (6 μm) of roots were obtained by cryosectioning. CD68 immunostaining was used to determine macrophage content, as described previously (44–46). Oil Red O immunostaining was used to determine neutral lipids. Moreover, Picrosirius red staining was used to assess the collagen content under polarized light. ImageJ 1.53t software (NIH) was used for all the quantifications.

### BCA analysis.

BCAs embedded in paraffin were sectioned (5 μm). Every fifth cross section was stained using the Movat’s pentachrome method to visualize atherosclerotic lesions. The maximal lesion site was determined, and adjacent sections were immunostained using a Mac-2 antibody (Cedarlane Lab, catalog CL8942AP; biotinylated secondary antibody goat-anti-rat, Southern Biotech, catalog 3050-08) to detect lesion macrophages, as described previously (47). We used a rat IgG2a isotype control antibody (Cedarlane Lab, catalog CLCR2A00) as a negative control for Mac-2 staining. Morphometric measurements were performed on digitized images of stained serial sections using Image-Pro Plus software (Media Cybernetics). At least 12 BCA sections per mouse were analyzed (60 sections/mouse with every fifth section used for Movat’s stain and morphometry), and the maximal lesion area value for each mouse was used as the summary parameter.

### Human recombinant APOB fragments.

Truncations of the human *APOB* gene to *APOB18* (3,279 bp, 90 kDa), *APOB15* (2,862 bp, 75 kDa), *APOB12* (2,454 bp, 60 kDa), *APOB8* (2,049 bp, 45 kDa), *APOB5* (1,638 bp, 30 kDa), and *APOB3* (1,234 bp, 15 kDa) were done using site-directed mutagenesis. APOB3 is too small for detection by the APOB antibody. For this, the primers (Supplemental Table 1) were designed using the NEBase changer online tool (https://nebasechanger.neb.com) in such a way that the amplified product retained only the truncated portion of APOB along with plasmid backbone. All truncated versions of APOB were amplified from pRP.ExTri-CMV-5′UTR-ApoB48. The amplified products were self-ligated and transformed in *E*. *coli* cells using the Q5 site-directed mutagenesis kit according to the manufacturer’s instructions (New England Biolabs, E0554). The plasmids containing truncated APOBs were isolated from recombinant *E*. *coli* transformants, and their correctness was confirmed by Sanger DNA sequencing.

The expression and secretion of these ApoB truncated proteins were verified in Cos-7 cells, as described previously (46). In brief, the Cos-7 cells were forward transfected with plasmids carrying APOB truncations. The media was changed after overnight incubation. After 24 hours, media was replaced with serum-free media. Media was collected after overnight incubation and centrifuged at 2,800*g* for 10 minutes to remove any dead cells or particulate matter. About 30 μL was resolved on 8% SDS-PAGE. The resolved protein was transblotted onto nitrocellulose membrane, and ApoB protein was immune detected using anti-APOB antibodies (Santa Cruz Biotechnology, sc-393636).

### Site-directed mutagenesis to generate APOB18 mutants.

The hAPOB18-FLAG plasmid was used as a template to create ApoB18 mutants using site-directed mutagenesis, and primers were designed using the NEBase charger tool (Supplemental Table 1). L645, L682, L687, W721, and F680 residues in APOB18 were substituted with Gly using the Q5 site-directed mutagenesis kit (New England Biolabs, E0554S), and mutations were confirmed by Sanger sequencing. Cos-7 cells were reverse transfected with 3 μg of plasmid expressing either WT APOB18-FLAG or mutant APOB18-FLAG and grown at 37°C in a humidified incubator. After 48 hours, cells were washed 3 times with PBS and incubated with fresh DMEM media without FBS. The overnight conditioned media was resolved on 8% SDS-PAGE, and ApoB-18 expression was detected using anti-FLAG M2 antibody (Sigma-Aldrich, catalog 3165). After confirming secretion of different fragments, the media was used for competition experiments.

### In vivo aortic endothelial uptake of DiI-labeled chylomicrons.

Mice (4 treated with control AAV and 4 with APOB18-AAV) were fasted overnight, and DiI-chylomicrons (0.5 mg TG/g BW) were injected retro-orbitally. Mice were euthanized 15 minutes after injection, and thoracic aortas and blood samples were collected for DiI-chylomicron uptake and plasma TG measurements. Aortas were fixed with 10% formalin, washed 3 times with PBS, and stained with DAPI to highlight nuclei. Confocal laser-scanning microscopy (Leica SP8) was used to observe the DiI-chylomicrons within aortic ECs. 5 randomly selected fields were acquired per sample, each containing a minimum of 50 ECs.

### Lipoprotein DiI labeling and isolation.

Lipoproteins from WT, *Apobec1^–/–^*, or APOB48-only mice were isolated by differential density centrifugation. For chylomicron isolation, plasma in a Beckman Coulter tube (catalog 344059) was overlaid with an equal volume of 1.006 g/cm^3^ density solution and centrifuged at 26,000*g* in a TH-641 swinging bucket rotor (Thermo Fisher Scientific) for 45 minutes at 12°C. The chylomicron layer at the top was transferred into an autoclaved 1.5 mL microcentrifuge tube using an autoclaved 9-inch Pasteur pipette. Following chylomicron isolation, the rest of the 1.006 g/cm^3^ density solution was aspirated, and the remaining plasma was overlaid with an equal volume of 1.12 g/cm^3^ density solution for LDL isolation. The mix was centrifuged at 26,000*g* for 22 hours at 12°C. The LDL layer at the top was transferred into an autoclaved 1.5 mL microcentrifuge tube using an autoclaved 9-inch Pasteur pipette.

When required, lipoprotein labeling with DiI was performed as described by Kraehling et al. (15). Briefly, DiI was dissolved in DMSO (3 mg/mL), and 10 μL of this solution was added per 250 μL of serum. This mixture was then incubated at 37°C overnight prior to lipoprotein isolation. The DiI-labeled lipoproteins were isolated and dialyzed against PBS containing 5.0 μM EDTA.

### In vitro knockdown of SR-BI and ALK1.

ECs were seeded in 0.1% gelatin-precoated 24-well plates (in glass coverslips, for downstream imaging studies), or 6-well plates (for Western blot assessment of SR-BI or ALK1 knockdown) and allowed to reach 40% confluency. The volumes below are for each well of a 6-well plate and could be scaled up or down according to experimental need. Before transfection with SR-BI ASO or ALK1 siRNA, culture medium was removed and cells were overlaid with 1.8 mL fresh, prewarmed culture medium with FBS. Then, 5 μL of a 10 μM ASO or siRNA stock solution was added to 95 μL of FBS-free DMEM in a sterile 1.5 mL Eppendorf tube, and 4 μL DharmaFECT 4 (Horizon Discovery) was added to 96 μL of DMEM in a separate sterile 1.5 mL Eppendorf tube. Both mixtures were combined by adding the DharmaFECT 4 mixture to the tube containing the ASO and were gently mixed by tapping the side of the tube. The transfection mixture was incubated at room temperature for 20 minutes. Cells were removed from the incubator, and 200 μL of the ASO/DharmaFECT 4 mix was added to each well of the 6-well plate. The mixture was delivered dropwise and evenly throughout each well. Cells were allowed to grow for 24 hours, then switched to FBS-free medium overnight. Knockdown of SR-BI was assessed by Western blot.

### Treatment with DiI-labeled lipoproteins in vitro.

Mouse aortic ECs (Angiocrine Biosciences) were seeded in glass coverslips precoated with 0.1% gelatin and allowed to reach 70% confluency. Before each experiment, cells were deprived of FBS overnight, and all experiments were carried out in FBS-free medium. Cells were exposed to DiI-labeled chylomicrons (4 mg/dL chylomicron TG) or LDL (2.5 μg/mL protein) in the absence or presence of APOB fragments as indicated, for 30 minutes at 37°C. Following treatment, cells were washed with PBS and fixed with 10% formalin for 20 minutes at room temperature. Nuclei were stained with DAPI. Image acquisition was performed by confocal laser-scanning microscopy (Leica SP8) of at least 4 independent experiments with 3 biological replicates. The area occupied by FLAG-APOB fluorescence was quantified using ImageJ/Fiji (NIH). A minimum of 4,000 cells were analyzed per condition.

### Membrane association studies.

Cells were cultured in glass coverslips, and knockdown of the receptors was performed as described above. Cells were allowed to reach confluency and exposed to FLAG-tagged APOB fragments for 10 minutes on ice to prevent internalization. Cells were then thoroughly washed with PBS to remove nonassociated fragments and fixed with 10% formalin. APOB fragments at the EC surface were immunostained using DYKDDDDK (FLAG) Tag Monoclonal Antibody (Invitrogen, catalog MA1-91878). Nuclei were stained with DAPI. Image acquisition was performed by confocal laser-scanning microscopy (Leica SP8) of 4 independent experiments with 3 biological replicates. The area occupied by FLAG-APOB fluorescence was quantified using ImageJ/Fiji. At least 4,000 cells were analyzed per condition.

### Image acquisition and analysis.

All imaging acquisition was performed at NYU Langone’s Microscopy Core. Samples were imaged by confocal laser-scanning microscopy (Leica SP8) in sequential scan mode with an HCX PL APO lambda blue ×63/1.40 oil objective lens at room temperature. DiI particle quantification was performed using ImageJ/Fiji.

### TIRF transcytosis assay.

Primary human coronary artery cells were purchased from PromoCell (catalog C12221). TIRF microscopy images were acquired on an Olympus cell TIRF Motorized Multicolor TIRF module mounted on an Olympus IX81 microscope. Samples were imaged using a ×150/1.45 objective with 561 nm excitation lasers and Volocity acquisition software. Unless otherwise indicated, the penetration depth, or the distance into the basal aspect of the cell that is visible by TIRF microscopy, was set at 110 nm. For each cell, 150 TIRF images were acquired at a frame rate of 6.67 per second for a constant duration of approximately 22 s. At least 10 randomly selected cells were imaged in each experimental replicate. DiI-labeled chylomicrons were added to confluent cells seeded on 25 mm glass coverslips. Cells were maintained at 4°C to allow binding; after 10 minutes, cells were rinsed in cold PBS and fresh media (HEPES-buffered RPMI). Samples were placed on the live-cell imaging stage at 37°C for 2 minutes before initial image acquisition using a standard cell chamber (Life Technologies, catalog A7816).

### Molecular modeling.

Predictions of APOB18-ligand complexes were carried out by AlphaFold2 using MMseqs2 available via ColabFold v1.5.5 (16) and using the web server: https://colab.research.google.com/github/sokrypton/ColabFold/blob/main/AlphaFold2.ipynb (commit ID 9712f2ff262d). The input consisted of the protein sequences of APOB18 (Uniprot ID P04114, residues 28–821, omitting the N-terminal signal sequence), SR-BI (Uniprot ID Q8WTV0, residues 33–552, omitting the N-terminal signal sequence), and ALK1 (Uniprot ID P37023, residues 23–503, omitting the N-terminal signal sequence), separated into 2 chains with the number of relaxed models set to 5 and no template mode. Calculations were carried out on the GPU provided by Google-Colab; all additional parameters were selected as default (msa_mode, mmseqs2_uniref_env; pair_mode, unpaired_paired; model_type, auto; num_recycles, 3; recycle_early_stop_tolerance, auto; relax_max_iterations, 200; pairing_strategy, greedy; max_msa, auto; num_seeds, 1).

### APOB48, APOB100, and APOB18 quantification by LC-MS/MS.

Total plasma proteins (10 μL) were denatured, reduced, alkylated, and digested with trypsin essentially following the protocol previously reported (48). Protein digests were then separated on an IonOpticks Aurora Elite 15 cm × 75 μm ID, 1.7 μm C18 column and Orbitrap Astral mass spectrometer in data-independent acquisition mode. To quantify total APOB (APOB-total), the 5 most abundant peptides from the sequence region 28–2,178 were selected; to quantify APOB100, the 5 most abundant peptides from the APOB sequence region 2,179–4,505 were chosen. Moreover, peptides homologous between mouse and human APOB sequence were excluded. Peptide intensities were summed to represent abundance of each of the forms. APOB48 was calculated as the difference between APOB-total and APOB100. Human APOB18 was quantified using the 5 most abundant peptides from the human APOB18 sequence (residues 28–809). Relative abundance of each form was then calculated as a percentage of APOB-total.

### APOB48, APOB100, and APOB18 quantification by ELISA.

Human APOB18 was measured in mouse plasma obtained from null and APOB18-AAV–injected mice groups following the protocol described for detection of APOB48 and APOB100 (49–51). Mouse monoclonal APOB antibody 1D1, which recognizes N-terminal amino acids 474–539 in human APOB, was used as capture antibody for the detection of APOB18. Mouse APOB48 and APOB100 in the plasma of those mice was detected using a commercial mouse APOB ELISA kit (Abcam, ab230932) according to the manufacturer’s instructions.

### Statistics.

Statistical analysis was performed using GraphPad Prism 7 software. Data are expressed as mean ± SD unless otherwise indicated. Measurements between 2 groups were performed with an unpaired 2-tailed Student’s *t* test. Groups of 3 or more were analyzed by 1-way ANOVA with Dunnett’s post hoc test as indicated. A *P* value of less than 0.05 was considered significant. Statistical parameters for each experiment can be found within the corresponding figure legends.

### Study approval.

All procedures were approved by the Institutional Animal Care and Use Committee at New York University Langone Health (animal protocol 160907, “Lipoprotein Lipase and ApoB”).

### Data availability.

All data supporting the findings of this study are provided in the main text, the supplementary materials, and the Supporting Data Values file.

## Author contributions

AGC planned and executed experiments. CC, WY, DN, JG, RK, and CH assisted with atherosclerosis studies. CC performed and analyzed confocal imaging studies. TWWH and WLL performed TIRF microscopy. NA, SV, and MMH created APOB fragments, performed site-directed mutagenesis, and measured APOB by ELISA. SR performed fast protein liquid chromatography. NOD created the APOB100 lipoproteins. TV measured APOB by LC-MS/MS. TM performed the molecular modeling. JEK and KEB did the BCA analysis. IJG, AGC, TM, EAF, NOD, KEB, and MMH planned experiments and participated in writing and editing.

## Conflict of interest

The authors have declared that no conflict of interest exists.

## Funding support

This work is the result of NIH funding, in whole or in part, and is subject to the NIH Public Access Policy. Through acceptance of this federal funding, the NIH has been given a right to make the work publicly available in PubMed Central.

- NIH grants HL164949 to IJG and MMH; P01HL151328 to IJG, NOD, KEB, JEK, and EAF; HL160470 to IJG, MMH, and EAF; HL158054 to MMH; R35HL150754 to KEB; and DK52574 and DK119437 to NOD.
- American Heart Association (postdoctoral fellowship award 830233 to AGC).
- Canadian Institutes of Health Research (PJT-168947 to WLL).
- Canada Research Chair in Mechanisms of Endothelial Permeability (to WLL).
- Ontario Graduate Scholarship (to TWWH).
- Queen Elizabeth II Graduate Scholarship in Science and Technology (to TWWH).
- Austrian Science Fund (FWF; P28854, I3792, DOC130, W1226, and COE14 to TM).
- Austrian Research Promotion Agency (FFG; 864690 and 870454 to TM).
- Integrative Metabolism Research Center Graz (to TM).
- Austrian Infrastructure Program 2016/2017 Styrian Government (Zukunftsfonds) (to TM).
- BioTechMed-Graz (Flagship project DYNIMO to TM).

## Supplementary Material

Supplemental data

Unedited blot and gel images

Supporting data values

## Version 1 04/23/2026

In-Press Preview

## Version 2 06/15/2026

Electronic publication
