# Supplementary material for: The N-terminus of apolipoprotein B mediates the interaction of atherogenic lipoproteins with endothelial cells
Source: J Clin Invest. 2026 Apr 23;136(12):e190513. doi: 10.1172/JCI190513 (PMC13262728; doi:10.1172/JCI190513)
Supplement: Supplemental data [file jci-136-190513-s235.pdf]

The N terminus of Apolipoprotein B mediates the interaction of atherogenic lipoproteins with endothelial cells

Ainara G. Cabodevilla[1], Camila Calistru[1], Waqas Younis[1], Dimitris Nasias[1], Tse W. W. Ho[2], Narasimha Anaganti[3], Swati Valmiki[3], Sujith Rajan[3], Jana Gjini[1], Rufina Kore[1], Carmen Hannemann[4], Nicholas O. Davidson[5], Tomas Vaisar[6], Jenny E. Kanter[6], Karin E. Bornfeldt[6,7], Edward A. Fisher[4], Warren L. Lee[2], Tobias Madl[8], M. Mahmood Hussain[3], Ira J. Goldberg[1*]

Corresponding author: Ira.Goldberg@nyulangone.org

The PDF file includes:

Figs. S1 to S4
Table S1

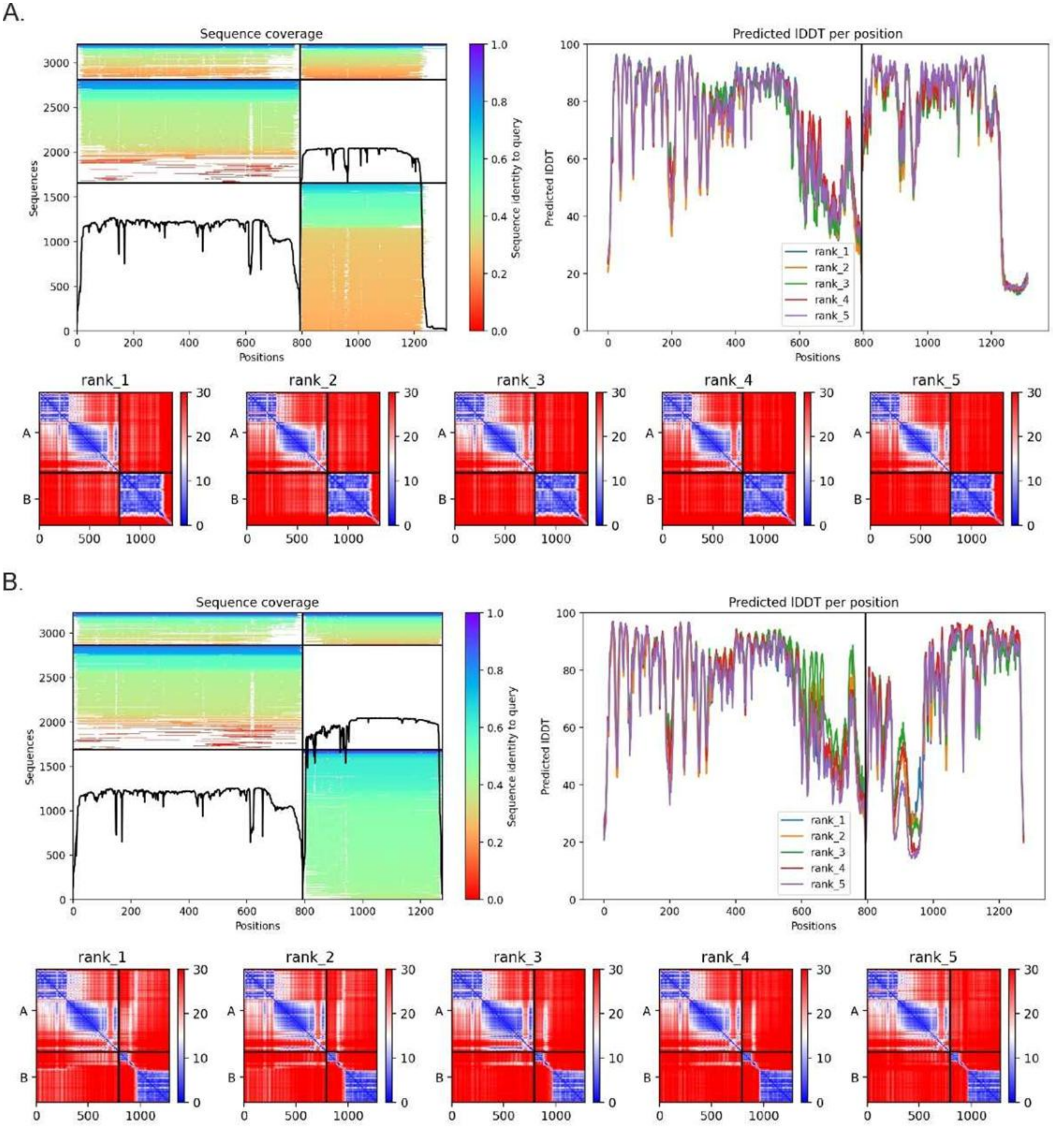

Fig. S1.

Summary of the AlphaFold Predictions for the binding of APOB18 with SR-B1 and ALK-1. Sequence coverage, predicted inter-residue distances, and predicted IDDT scores for the APOB18-SR-BI (**A**) and APOB18-ALK1 (**B**) complexes. Sequence coverage showing the alignment of multiple sequence alignments (MSAs) across the input sequences. The heatmap

represents sequence identity, ranging from low (red) to high (blue). The black line indicates the effective sequence coverage. Predicted IDDT (Local Distance Difference Test) per position plotted against the sequence positions. The IDDT values are plotted for the top five ranked predicted models, showing overall structural confidence across positions. Predicted inter-residue distance maps for the top five ranked models. The heatmaps illustrate pairwise residue distances, with red indicating close distances and blue indicating larger distances. Models are labeled as rank_1 through rank_5, corresponding to different AlphaFold confidence scores.

A.

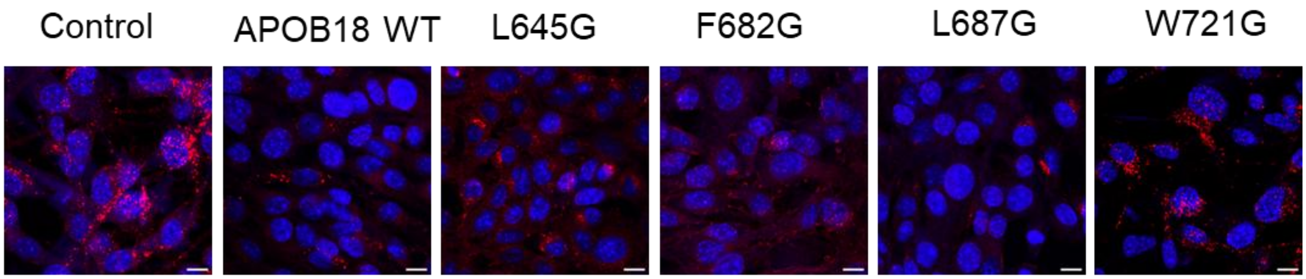

B.

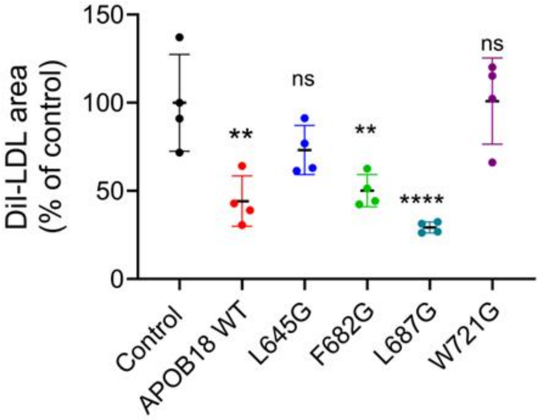

Fig. S2.

L645G and W721G APOB18 mutant fragments fail to inhibit EC LDL uptake. ECs were treated with excess (25 mg/dL) unlabeled LDL in serum-free medium overnight to deplete LDLR, then incubated with DiI-labeled LDL (2.5 mg/dL) in serum-free medium and in the presence of control medium or media obtained from Cos-7 cells transfected with plasmids expressing WT apoB18 or different indicated mutants. DiI-LDL uptake was significantly and similarly inhibited by wild type, F682G and L682G APOB18. L645G and 2721G APOB18 did not significantly block the uptake of DiI-LDL. Scale bar 20μM. **p<0.001, ****p<0.00001, one-way ANOVA followed by Dunnett's post hoc multiple comparisons test against control.

A.

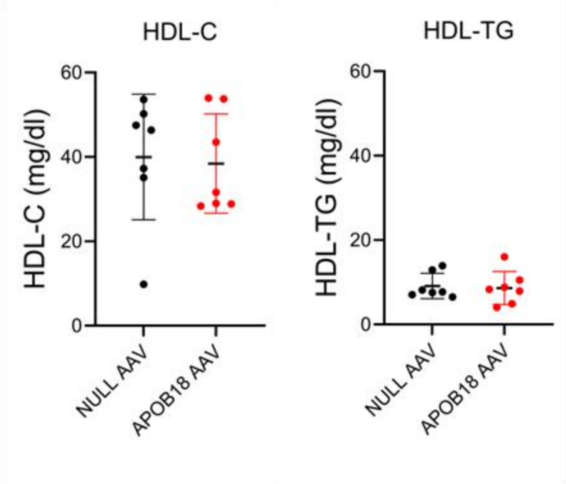
HDL-C
HDL-C (mg/dl)
60
40
20
0
NULL AAV
APOB18 AAV
HDL-TG
HDL-TG (mg/dl)
60
40
20
0
NULL AAV
APOB18 AAV

B.

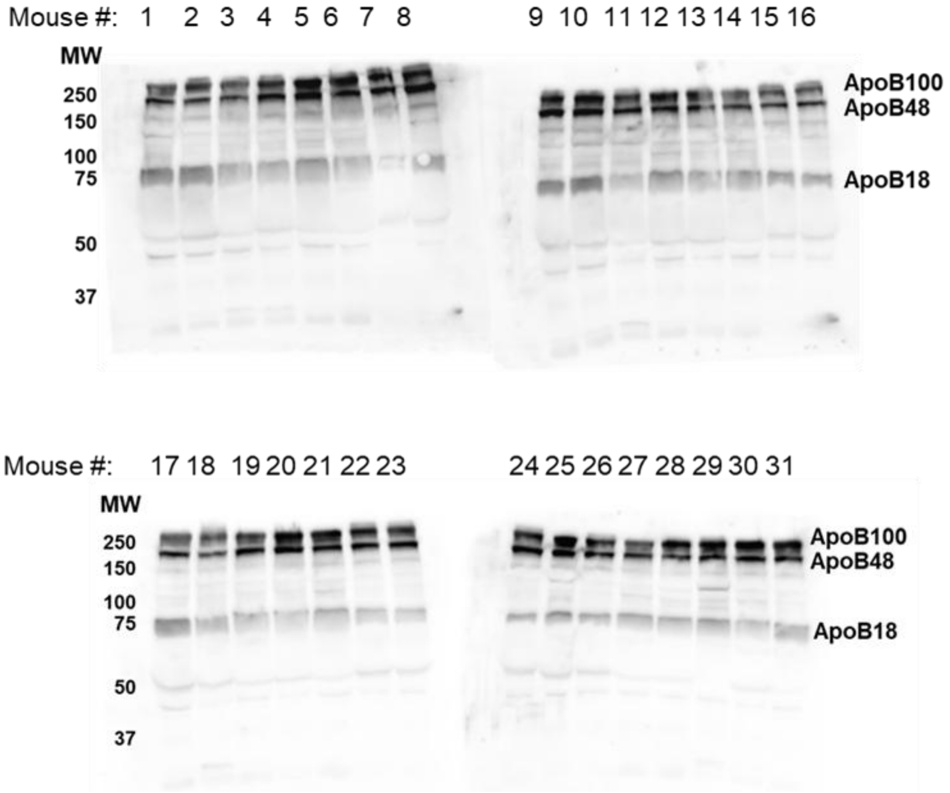
Mouse #: 1 2 3 4 5 6 7 8
9 10 11 12 13 14 15 16
MW
250
150
100
75
50
37
ApoB100
ApoB48
ApoB18
Mouse #: 17 18 19 20 21 22 23
24 25 26 27 28 29 30 31
MW
250
150
100
75
50
37
ApoB100
ApoB48
ApoB18

C.

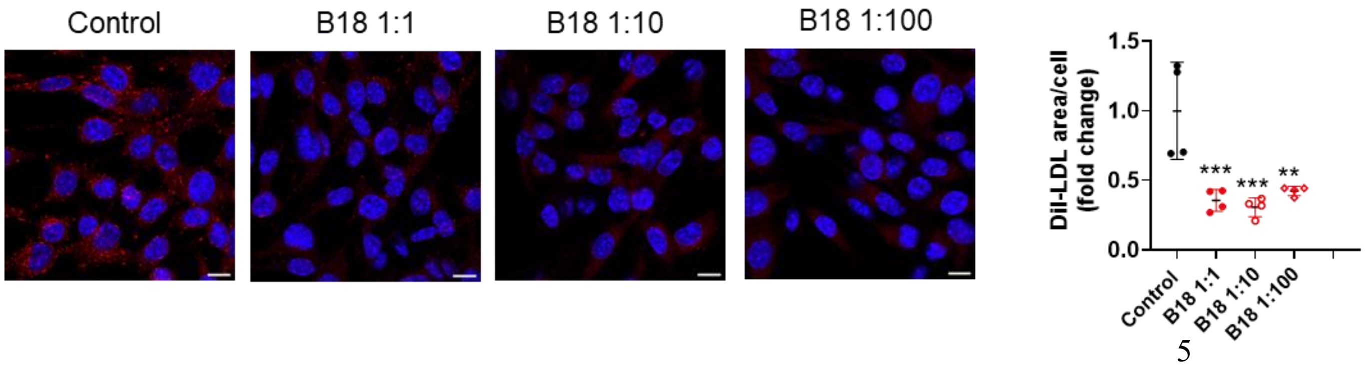
Control
B18 1:1
B18 1:10
B18 1:100
DiI-LDL area/cell
(fold change)
1.5
1.0
0.5
0.0
***
***
**
Control
B18 1:1
B18 1:10
B18 1:100

Fig S3.

APOB18 inhibits uptake of LDL. A. *Ldlr*$^{-/-}$ mice treated with control (null) or APOB18 AAV have similar levels of circulating HDL cholesterol (left panel) and triglyceride (right panel) following 12 weeks on a western diet. **B**. Immunoblot shows APOB18 expression in the plasma of APOB18 TBG-AAV8 treated mice. C. Uptake of DiI-LDL by ECs is significantly inhibited by APOB18 at relative concentrations of 1:1, 1:10, and 1:100. **$p<0.001$, ***$p<0.0001$, one-way ANOVA followed by Dunnett's post hoc multiple comparisons test against control.

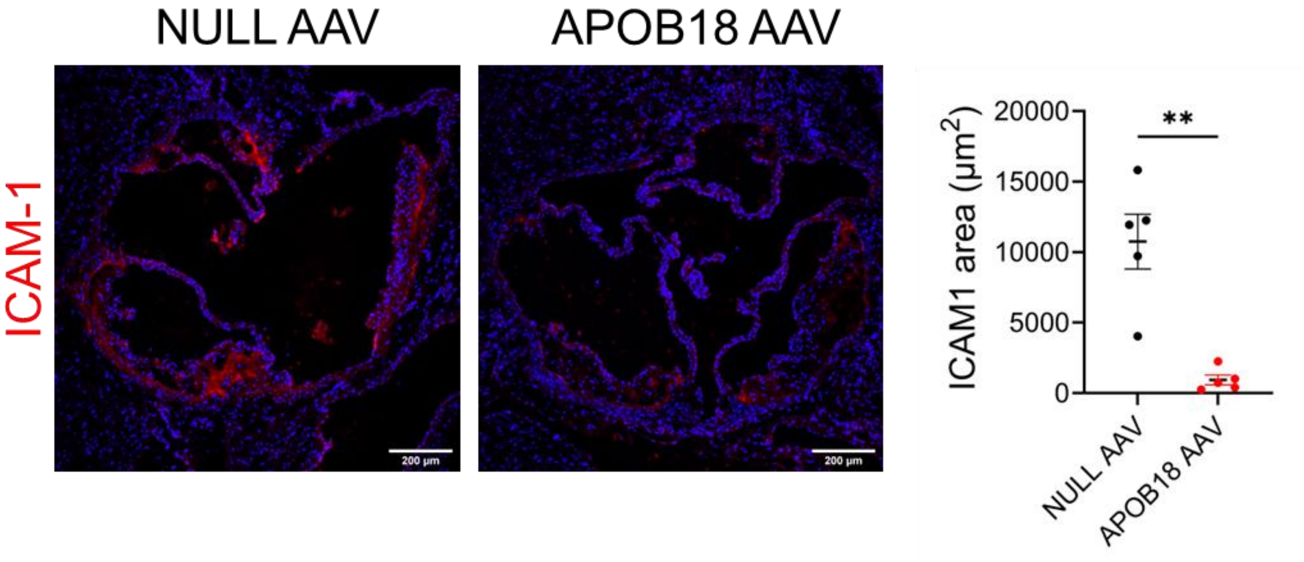

Fig. S4.

APOB18-AAV reduces expression of ICAM-1 in aortic roots of *Ldlr*$^{-/-}$ mice. Expression of ICAM-1 in the aortic roots was assessed in a subset of mice (5 per group) and was found to be significantly reduced in APOB18-AAV treated mice. **p<0.001, unpaired Student's t test.

**Primer**	**5'---------->3'**
ApoB-3_F	TAAACCCAGCTTTCTTGTAC
ApoB-3_R	TTCTGGAATGGCCAGCTT
ApoB-6_R	CTTGTAGGAGAAAGGCAG
ApoB-9-R	CACATCTATCAGAAGGGG
ApoB-12-R	AAGAAGAACCTCCTGGTC
ApoB-15-R	TCCAAAGGCAGTGAGGGT
ApoB-18-R	GCCCTTCCTGATGACCTC
L645G-FP	TCTGTTTCTGGTCCATCACTTGACC
L645G-RP	TTTGTAGAGTTGATAGTTCC
F680G-FP	CTCACTGCCGGTGGATTTGCTTC
F680G-RP	GGTAGTTTTCAGCATGC

Table S1.
Primers used for the truncation of APOB
