# Supplementary figures and images for: The N-terminus of apolipoprotein B mediates the interaction of atherogenic lipoproteins with endothelial cells
Source: J Clin Invest. 2026 Apr 23;136(12):e190513. doi: 10.1172/JCI190513 (PMC13262728; doi:10.1172/JCI190513)

Figure 1B

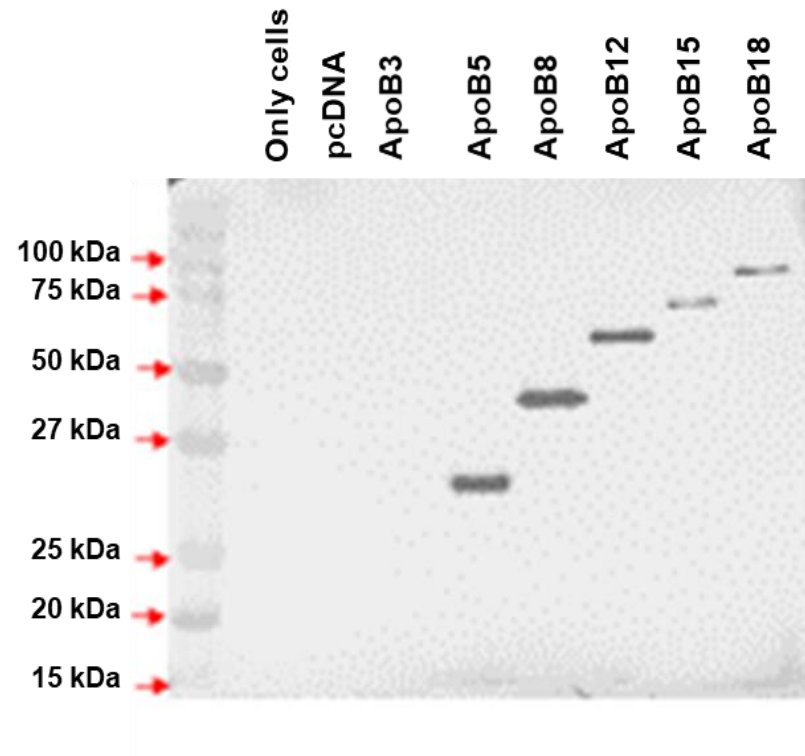

Figure 1G

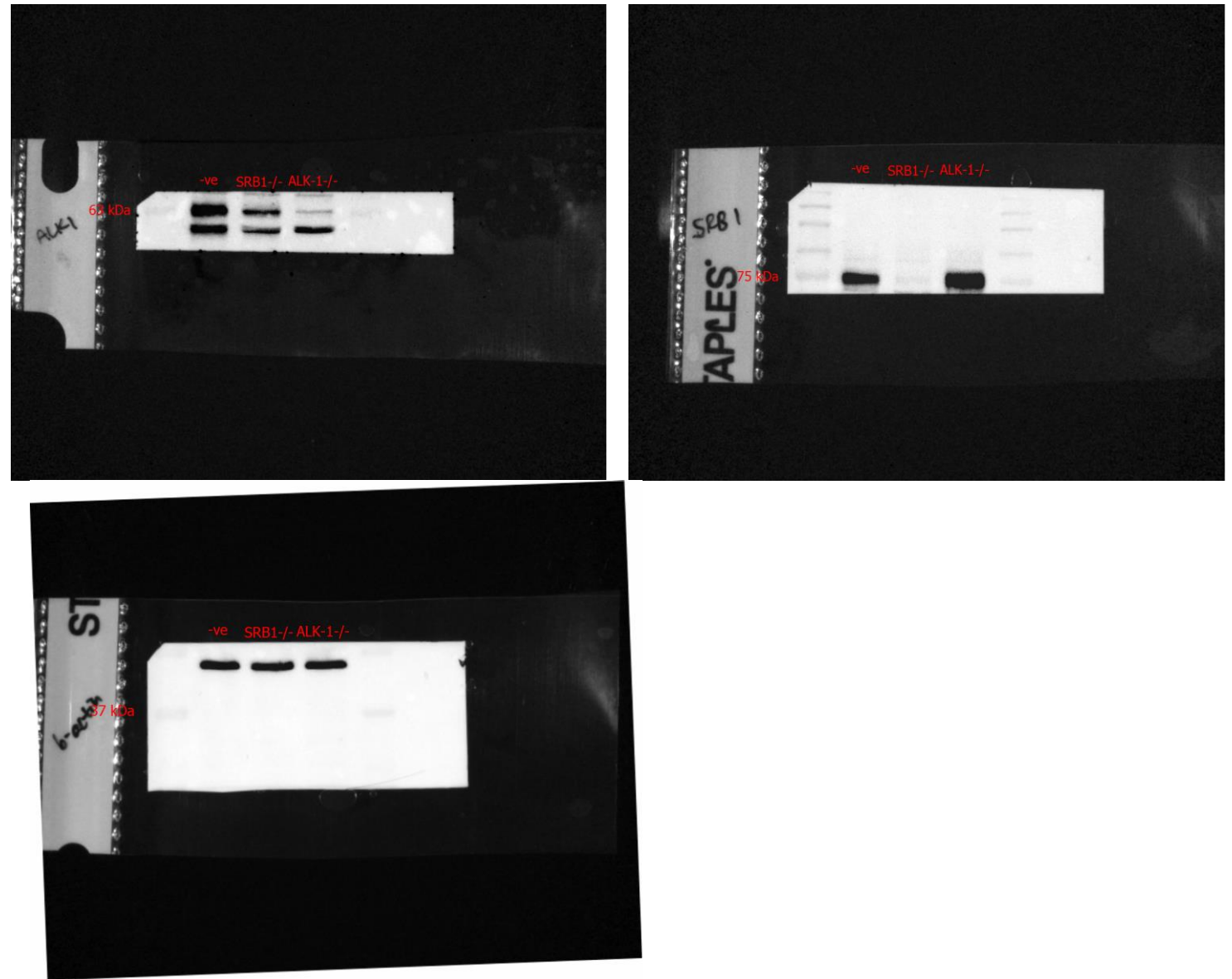

Figure 5F

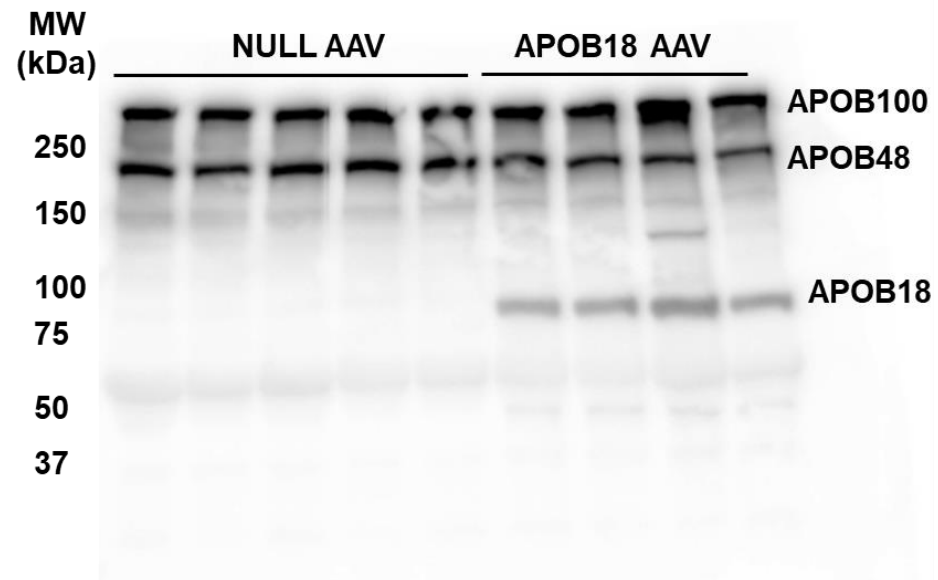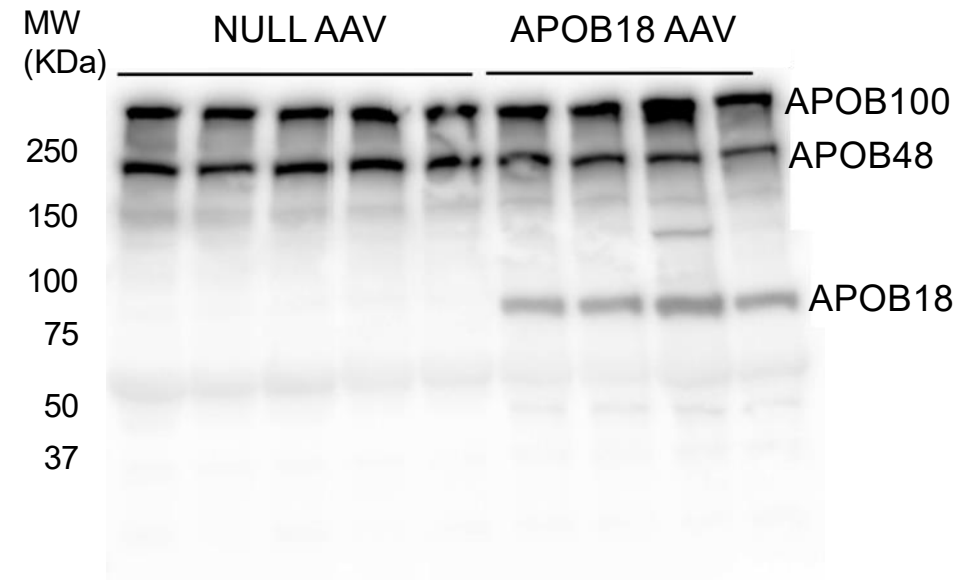

Figure S3B

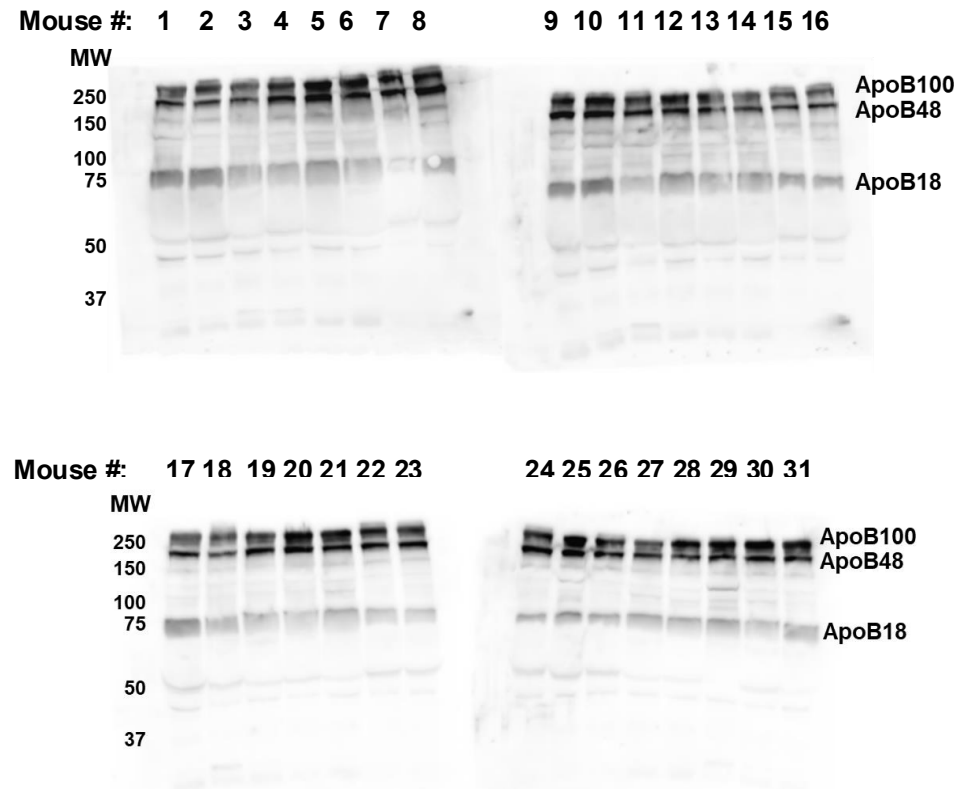

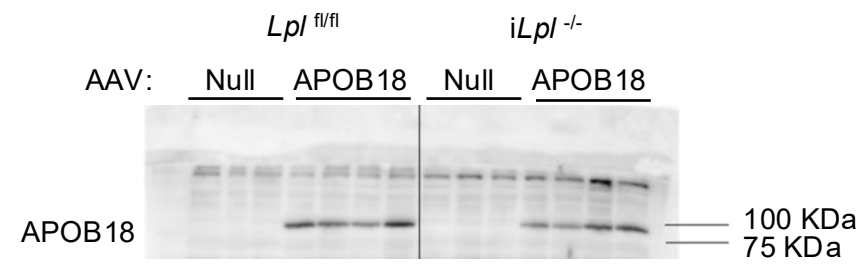

Supplement: Unedited blot and gel images [file jci-136-190513-s236.pdf]
